# To Divide or Not to Divide? How Deuterium Affects Growth and Division of *Chlamydomonas reinhardtii*

**DOI:** 10.3390/biom11060861

**Published:** 2021-06-09

**Authors:** Veronika Kselíková, Vilém Zachleder, Kateřina Bišová

**Affiliations:** 1 Laboratory of Cell Cycles of Algae, Centre Algatech, Institute of Microbiology of the Czech Academy of Sciences, 37981 Třeboň, Czech Republic; kselikova@alga.cz (V.K.); zachleder@alga.cz (V.Z.); 2 Faculty of Science, University of South Bohemia, 37005 České Budějovice, Czech Republic

**Keywords:** deuterium, heavy water, cell cycle, multiple fission, commitment point, stress, cell division, *Chlamydomonas reinhardtii*

## Abstract

Extensive in vivo replacement of hydrogen by deuterium, a stable isotope of hydrogen, induces a distinct stress response, reduces cell growth and impairs cell division in various organisms. Microalgae, including *Chlamydomonas reinhardtii*, a well-established model organism in cell cycle studies, are no exception. *Chlamydomonas reinhardtii*, a green unicellular alga of the *Chlorophyceae* class, divides by multiple fission, grows autotrophically and can be synchronized by alternating light/dark regimes; this makes it a model of first choice to discriminate the effect of deuterium on growth and/or division. Here, we investigate the effects of high doses of deuterium on cell cycle progression in *C. reinhardtii*. Synchronous cultures of *C. reinhardtii* were cultivated in growth medium containing 70 or 90% D_2_O. We characterize specific deuterium-induced shifts in attainment of commitment points during growth and/or division of *C. reinhardtii*, contradicting the role of the “sizer” in regulating the cell cycle. Consequently, impaired cell cycle progression in deuterated cultures causes (over)accumulation of starch and lipids, suggesting a promising potential for microalgae to produce deuterated organic compounds.

## 1. Introduction

Deuterium (^2^H or D) is a stable isotope of hydrogen with a natural abundance of 0.015% [1]. The biggest and only difference between the atomic structures of deuterium and protium (^1^H) is the extra neutron in the deuterium nucleus. This change in the atomic structure results in unprecedented consequences in its physico-chemical characteristics. Heavier isotopes generally exhibit slower reaction rates, with the phenomenon being described as the kinetic isotope effect. This effect is normally very low [2], but not so with deuterium. As deuterium exhibits the largest relative increase in atomic mass compared to its lighter isotope protium, it also exhibits the strongest kinetic isotope effect observable among stable isotopes of biogenic elements [3]. Exposing living organisms to stable isotopes of other biogenic elements (^13^C, ^15^N, ^17^O or ^18^O) has little or no effect [3]; however, their exposure to deuterium results in various physiological and morphological aberrations. Deuterium disrupts bio-signaling [4] and bio-energetic processes both in mitochondria [5,6] and chloroplasts [7,8]. One of the most pronounced effects of deuterium is the slowing or disruption of growth and cell division in deuterated cells and organisms. This phenomenon has been observed in animals [9,10] and plants [11,12], as well as bacteria [13,14]. Nevertheless, growing microorganisms on deuterated substrates still represents a promising non-synthetic way of obtaining highly deuterated compounds with minimal inputs [15,16,17].

**Figure 1:**
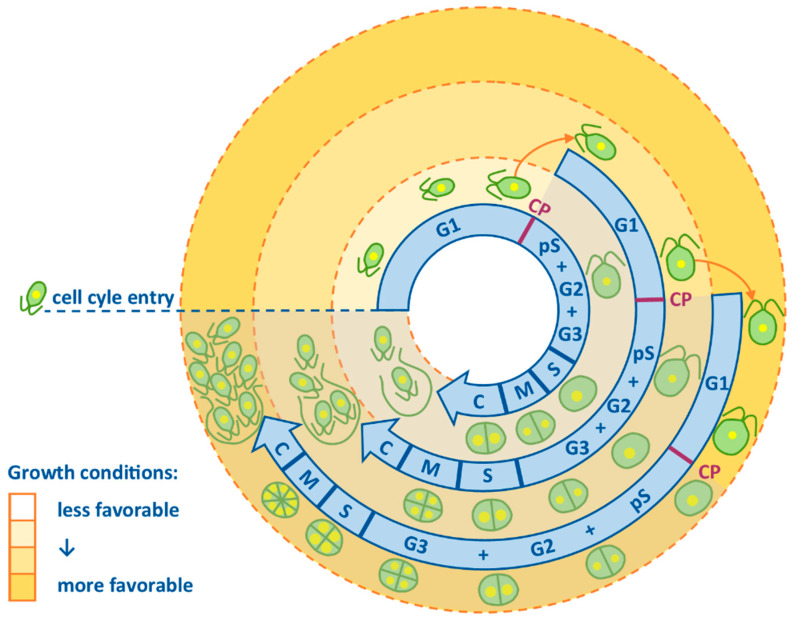
Multiple fission cell cycle in the model alga *Chlamydomonas reinhardtii*. Multiple overlapping growth and DNA replication–division sequences within one cell cycle are demonstrated as individual circle-shaped arrows. The better the growth conditions, the more growth and DNA replication–division sequences will be executed. The innermost ring represents division into two daughter cells. The combination of the innermost and the middle rings will lead to division into four daughter cells. Combination of all the circles will produce eight daughter cells. Each growth and DNA replication–division sequence can be further divided into several phases: G1 characterized by growth of the cell; then cells reach commitment point (CP) characterized by gaining the ability to divide even without further energy supply (shown by the shaded part after CP was reached); pS, the pre-replication phase; S phase during which DNA is replicated; G2 phase spanning from termination of DNA replication to the start of mitosis; M phase during which nucleus is divided; G3 phase between nuclear and cellular division and C phase, when the cell is cleaved. Adapted with permission from Bisova and Zachleder [25]. Copyright 2014 Oxford University Press, on behalf of the Society for Experimental Biology.

The ability (or more often the inability) of various organisms to grow in a deuterium-enriched environment has fascinated researchers since the 1930s [18,19]. These early studies were usually unable to distinguish between two separate yet interconnected processes in the cell—growth and division—which may be affected by deuterium to very different extents. The way to overcome this methodological problem is to choose a model organism that allows a simple separation of growth and cell division. One such class of organism is green alga which, when grown autotrophically, uses light as a source of energy for growth and division. While growth is light-dependent, division itself is light-independent. This feature of green algae is an advantage when used for preparing synchronous populations, i.e., populations in which the majority of cells are in the same stage of their cell cycle. High degrees of synchrony can be attained by simple alternating light and dark regimes of different lengths [20]. This enables effective separation of growth and reproductive events. One well-established model green alga is *Chlamydomonas reinhardtii*, a unicellular alga with two flagella, multiple mitochondria and a single cup-shaped chloroplast. *C. reinhardtii* is of special importance in cell cycle studies because it divides by multiple fission. In the most common type of cell division, binary fission, the cell cycle would follow a sequence of events: growth (G1 phase), synthesis of essential biomolecules including DNA replication and division (S phase), further growth (G2 phase) and nuclear division followed by cytokinesis into 2 daughter cells (M phase) [21]. During multiple fission, a single mother cell divides into 2*^n^* daughter cells, where *n* is the number of doublings. For *C. reinhardtii,* the most typical number of daughter cells is eight (*n* = 3) at optimal growth conditions [22] and decreases to four (*n* = 2) or two (*n* = 1) in less than optimal conditions [23,24]. This is possible thanks to an overlap in several growth and DNA replication and division sequences within one cycle, which are followed by cytokinesis at the end of the cycle (Figure 1). The G1 phase needs to be long enough to ensure sufficient growth and energy reserves for the upcoming division events. The threshold is governed by the ability of the cell to divide without further energy supply, i.e., in autotrophic organisms if the light supply is stopped. This threshold is reached at the commitment point (CP) in plants and algae. Several CPs are attained within one cell cycle in the multiple fission model. The existence of several CPs allows a maximization of growth as long as the energy source (in autotrophy, light) is available and a postponement of cell division to a time when no external energy source is available. This creates conditions for considerable plasticity that is not only beneficial for the cells but at the same time provides excellent tools for studying the cell cycle. 

## 2. Materials and Methods

### 2.1. Organism, Culture Growth Conditions

*Chlamydomonas reinhardtii* strain 21gr (CC-1690; Chlamydomonas Genetics Center, Duke University, Durham, NC, USA) was cultivated in glass cylinders with an inner diameter of 3 cm and volume of 300 mL. The cylinders were placed in a temperature-controlled water bath and illuminated by incandescent lamps (Dulux L55W/950 daylight, Osram, Munich, Germany). Prior to the experiment, cell cultures were synchronized using alternating light (13 h) and dark (11 h) regimes for at least three cycles. During synchronization, growth conditions were: 30 °C, bubbling with 2% (*v*/*v*) CO_2_ in air, incident light intensity of 500 μmol·m^−2^·s^−1^ and cultures were grown in modified HS medium as described by Hlavová, Vítová and Bišová [20]. Synchronized cultures were gently centrifuged at 3000× *g* for 5 min and re-suspended either in HS medium as described above or in HS medium containing 70% deuterium oxide (D_2_O) or 90% D_2_O (99.95 atom%, catalog number 300101500, Silantes, Munich, Germany). The growth conditions during experiments were the same as during synchronization except for light intensity, which was reduced to 200 μmol·m^−2^·s^−1^. Cultures grown in 0% D_2_O HS medium, 70% D_2_O HS medium or 90% D_2_O HS medium were sampled in 2 h, 4 h or 6 h intervals, respectively.

### 2.2. Assessment of Growth 

To determine culture growth, dry mass and optical density were evaluated. For dry mass, 5 mL of the culture were pelleted in pre-weighed tubes at 5000× *g* for 3 min and the supernatant was removed. The pellet was then dried at 100 °C to a constant weight. Optical density was measured at 750 nm. To assess cell counts as well as cell volumes (representing cellular growth), 1 mL of the culture was fixed with 2.5% (*w*/*v*) glutaraldehyde to a final concentration of 0.25% (*w*/*v*). These samples were then diluted 20 to 50 times with 0.9% (*w*/*v*) NaCl to a final volume of 10 mL and measured using a Multisizer 4 (Beckman Coulter, Brea, CA, USA). Cell volume was presented as modal value, i.e., the most common value in the dataset. This representation better reflects the central tendency of the cell size in a population with wide and skewed distribution. Cell division was assessed manually using light microscope. 

### 2.3. Analysis of Commitment Point Attainment

Sampled cultures were diluted 5 times and 10 times using their respective HS media in order to ensure an appropriate cell density. Three microliters of both dilutions were spotted onto plates containing HS medium solidified with 1.5% agar. Every sample was plated on three different plates, containing 0% D_2_O, 70% D_2_O and 90% D_2_O (Table 1). All plates were incubated at 30 °C in the dark for a minimum of 12 h (exact time was determined for every plate by observation of dividing cells). Plates were fixed bottom-up by adding a drop of Lugol’s solution (5% KI, 2.5% I) onto the lid of the plate. Fixed plates were stored at 4 °C until analysis. The number of undivided mother cells and mother cells that divided into two, four or eight daughter cells was then counted using a light microscope. Commitment curves represent the cumulative percentage of cells that divided to two, four or eight daughter cells as a function of sampling time. For the detailed protocol, see Kselíková et al. [26]. 

### 2.4. Kinase Assay

Cultures containing 2 × 10^7^ cells were pelleted, washed with SCE buffer (100 nM sodium citrate, 2.7 mM sodium EDTA, adjusted to pH 7 with citric acid), frozen in liquid nitrogen and stored at −70 °C until analysis. Protein lysates were prepared from pelleted samples as described by Bisova et al. [27], for details see Bišová [28]. The lysates were either used directly for the kinase assay or were purified with CrCKS1 beads [27]. Histone H1 kinase activity was measured in a final volume of 10 μL with either 7 μL of whole cell lysate or the CrCKS1 purified fraction following the protocol of Langan et al. [29]. The master mix consisted of 20 mM HEPES, pH 7.5, 15 mM MgCl_2_, 5 mM EGTA, 1 mM DTT, 0.1 mM ATP, 0.2% (*w*/*v*) histone (cat. number H5505, Sigma-Aldrich, St. Luis, MO, USA) and 0.37 MBq (γ ^32^P) ATP. The reactions were carried out at room temperature for 30 min and then stopped by adding 5 μL of 5 × SDS sample buffer (250 mM Tris-HCl, pH 6.8, 50% (*v*/*v*) glycerol, 10% (*w*/*v*) SDS, 100 mM DTT, 0.5% (*w*/*v*) bromophenol blue) and incubated for 2 min at 98 °C. Proteins were separated by SDS-PAGE in 15% gels [30]. Phosphorylated histone bands were visualized by autoradiography and analyzed using a phosphoimager (Storm 860, Molecular Dynamics, Chatsworth, CA, USA). Quantification of phosphorylation was achieved using Image Studio Lite software (ver. 5.2, LI-COR Biosciences, Lincoln, NE, USA). To ensure comparability between samples and experiments, the sums of pixel intensities within the same area were normalized to the background pixel intensity to yield the pixel intensity of the signal. These were further normalized to the pixel intensity of histone bands in the gels stained with Coomassie Brilliant Blue. Resulting values are presented as the sum of pixel intensity [31].

### 2.5. Quantification of Starch Content

Quantification based on the method of McCready et al. [32] was performed as published previously [33], with modification of the extraction procedure as described below. Two mL of an algal suspension was harvested by centrifugation at 18,000× *g* for 2 min and pellets were frozen at −20 °C. Algal cells were disintegrated by vortexing with 300 μL of glass beads in 500 μL of distilled water. Pigment extraction was done three times in 1 mL of 80% ethanol for 15 min at 68 °C. Each round was followed by centrifugation and removal of the supernatant. For total starch hydrolysis, 1.5 mL of 30% perchloric acid was added to the sediment, the samples were mixed by vortexing and then incubated at room temperature for 15 min. The samples were then centrifuged, and the supernatant was collected. This procedure was repeated three times, yielding 4.5 mL of hydrolyzed starch extract, which was then made up to a total volume of 5 mL. Starch determination with anthrone solution followed the original protocol [33].

### 2.6. Measurement of Neutral Lipid Content 

Neutral lipid content was measured spectrophotometrically in a microplate format following the modified procedure of Takeshita et al. [34]. Aliquots (100 μL) of the cultures were transferred to a 96-well plate and 5 μL of freshly prepared Nile Red dye (0.5 mg/mL in DMSO, catalog no. 72485, Sigma-Aldrich, St. Luis, MO, USA) were added to each well. The same amount of Nile Red dye was added to a sample blank consisting of 100 μL of H_2_O. The plate was incubated at room temperature for 15 min. Fluorescence was measured using an Infinite 200 PRO microplate reader (Tecan, Männedorf, Switzerland) equipped with a 485 nm excitation filter and a 595 nm emission filter. Fluorescence intensity of the samples was normalized using fluorescence intensity of unstained samples and a blank. Standard curves produced from a commercial lipid standard, triolein (catalog no. Y0001113, Sigma-Aldrich, St. Luis, MO, USA) were used to quantify neutral lipids.

### 2.7. Statistical Analysis 

Experiments were performed in three biological replicates. If not stated otherwise, all results are presented as an average and standard deviation (*n* = 3). The modal value was selected to characterize cell volume distribution in the population, as it corresponds to the most common value in the dataset. 

## 3. Results

### 3.1. Growth of Cultures in Medium Containing D_2_O

**Figure 2:**
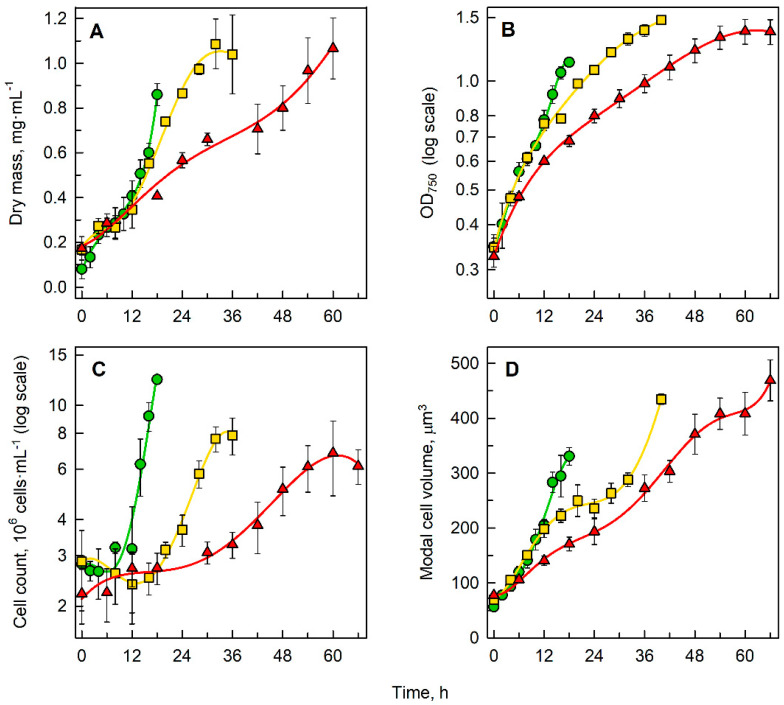
Changes in dry mass (**A**), optical density at 750 nm (**B**), cell count (**C**) and cell volume (**D**) of *C. reinhardtii* grown in 0% D_2_O (green circles), 70% D_2_O (yellow squares) or 90% D_2_O (red triangles). All cultures were grown and sampled for the duration of one cell cycle. OD: optical density.

Cells were synchronized by alternating light/dark regimes prior to the experiment and then grown in continuous light for one cell cycle, i.e., from their birth until completion of cell division. However, some experimental treatments affected the ability of cells to complete their cell division (see below). In such cases, the time point corresponding to cell division maxima was considered to be the end of cell cycle. Cells failing to divide until this point rarely divided later (data not shown). The cell cycle lengths for cultures grown in 0% D_2_O, 70% D_2_O and 90% D_2_O were established as 16, 36 and 60 h, respectively. The growth of each culture was monitored only during this time span. All the cultures arose from the same initial culture and started from approximately the same cell density. During one cell cycle, the cultures attained a dry mass of 0.85 mg mL^−1^ in 0% D_2_O, 1.05 mg mL^−1^ in 70% D_2_O and 1.08 mg mL^−1^ in 90% D_2_O (Figure 2A). In terms of optical density at 750 nm, the experimental treatments differed in the same manner, reaching maximal values of 1.12 in medium containing 0% D_2_O, 1.48 in 70% D_2_O and 1.37 in 90% D_2_O (Figure 2B). Significant differences in cell counts were also noted, where in 0% D_2_O the culture attained 12.3 × 10^6^ cells mL^−1^ after one cell cycle, i.e., five times the initial cell number, while in 70% D_2_O the cell number was less, by approximately one-third, than that in 0% D_2_O, reaching 7.9 × 10^6^ cells mL^−1^, i.e., nearly three times the initial cell number. In 90% D_2_O, the cell number after one cell cycle was even less, reaching a maximum of 6.8 ×10^6^ cells mL^−1^ corresponding to three times the initial cell number. While the cell count decreased with increasing D_2_O concentration, the cell volume followed an inverse trend. In 0% D_2_O, cells reached a modal cell volume of 330 μm^3^ at the time of cell division, while in 70% D_2_O, the modal cell volume was 430 μm^3^, and in 90% D_2_O, the modal cell volume was 470 μm^3^ at the time of cell division. The modal cell volume represents a typical cell of the population rather than an average cell. No drop in modal cell volume was observed, because the majority of daughter cells stayed in the division clusters at the end of the experiment and did not immediately release from the mother cell wall. The daughter cells of the control cultures released from the division cluster over several hours in light or in dark. The cells of all of the deuterated cultures stayed in the division clusters for a prolonged period of time (over 10 h). The observed cell numbers and cell volumes pointed to the fact that with increasing D_2_O concentrations, the cells grew larger and the cultures attained lower cell numbers. 

### 3.2. Accumulation of Energy Storing Compounds

**Figure 3:**
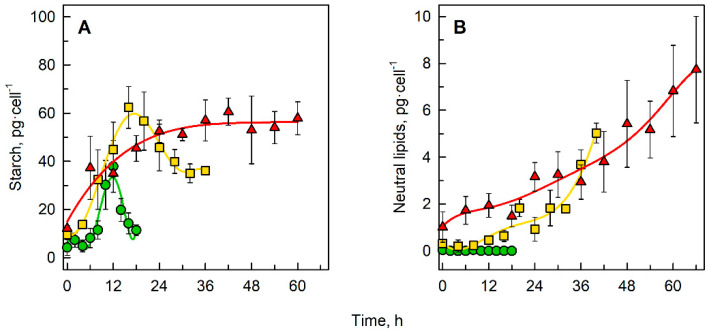
Changes in starch content per cell (**A**) and neutral lipid content per cell (**B**) in synchronous cultures of *C. reinhardtii* grown in 0% D_2_O (green circles), 70% D_2_O (yellow squares) or 90% D_2_O (red triangles).

The cellular content of energy storing molecules such as starch (Figure 3A) and neutral lipids (Figure 3B) changed with cell cycle progression. The starch content in a synchronous culture of *C. reinhardtii* grown in 0% D_2_O reached a maximum of 38 pg·cell^−1^ in the twelfth hour of the cell cycle (Figure 3A). Then the starch content decreased to 11 pg·cell^−1^ as the cells in the culture divided. In the culture grown in 70% D_2_O, the starch content increased until the sixteenth hour of the cell cycle, when it reached a maximum of 62 pg·cell^−1^, representing 163% of the value in the control culture, and then decreased to 35 pg·cell^−1^ with further progression of the cell cycle (Figure 3A). In the culture grown in 90% D_2_O, the starch content per cell increased gradually for approximately 24 h, then it reached a plateau at 56 pg·cell^−1^, representing 147% of the value of the control culture, and did not change any further until the end of the cell cycle (Figure 3A). Different D_2_O concentrations in the growth medium also affected the neutral lipid content in the cells (Figure 3B). In 0% D_2_O there was no change in neutral lipid content throughout the cell cycle, while both in 70% and 90% D_2_O, the lipid content increased considerably with cell cycle progression, reaching a maximum at the end of the cell cycle. In 70% D_2_O the maximum neutral lipid content was 5.0 pg·cell^−1^, in 90% D_2_O it was 7.7 pg·cell^−1^.

### 3.3. Kinase Activity and Cell Division

**Figure 4:**
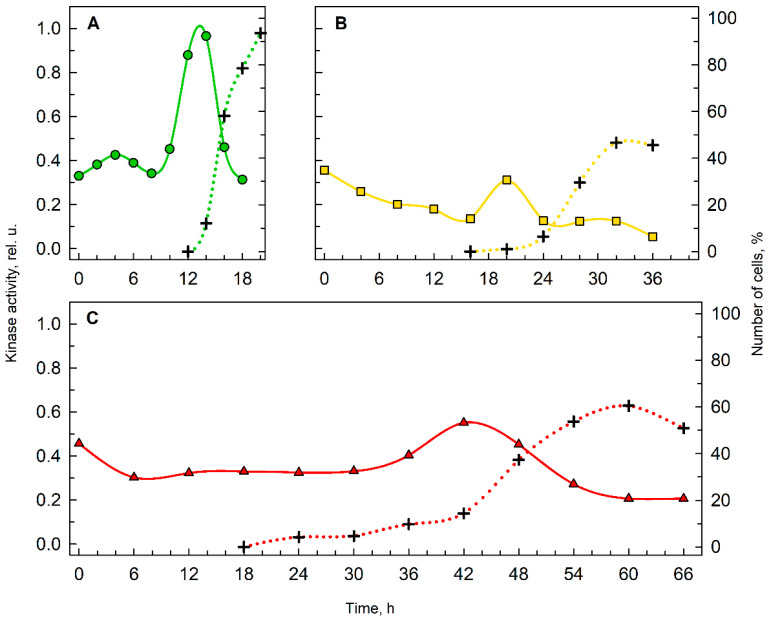
Histone H1 kinase activity in cultures grown in 0% D_2_O (**A**), 70% D_2_O (**B**) and 90% D_2_O (**C**). Kinase activity (solid line, left axis) precedes cell division (dotted line, right axis). One representative replica of the triplicate is shown for each condition.

To understand cell division in deuterated cultures, the proportions of cells completing cell divisions were calculated and the kinase activities of the key cell cycle regulator, cyclin-dependent kinase, were established (Figure 4). In medium with 0% D_2_O (Figure 4A), 95% of the cells performed cell division, but in 70% D_2_O (Figure 4B) and 90% D_2_O (Figure 4C) the proportions of cells able to complete cell division were reduced, reaching a maximum of 45% and 60% of the cells, respectively. In terms of histone H1 kinase activity, in the control culture with 0% D_2_O, activity increased rapidly prior to cell division, while in both deuterated cultures, the increase in kinase activity was slower and less significant. Nevertheless, the lower kinase activity in deuterated cultures corresponded to the lower proportion of cells finishing their cell division. 

### 3.4. The Effect of D_2_O on Commitment Point Attainment 

**Figure 5:**
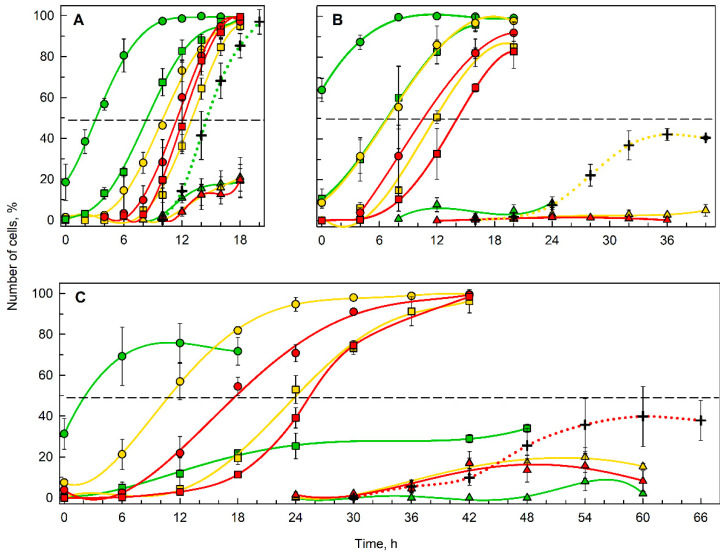
Percentage of cells attaining commitment point (CP) during growth in control medium with 0% D_2_O (**A**), in medium with 70% D_2_O (**B**) and in medium with 90% D_2_O (**C**). Each of the graphs shows CP attainment as the percentage of cells that finished cell division in the dark on plates with 0% D_2_O (green lines), 70% D_2_O (yellow lines) and 90% D_2_O (red lines). Attainment of the first CP (circles), the second CP (squares) and the third CP (triangles) is shown individually as analyzed from plates with 0% D_2_O, 70% D_2_O or 90% D_2_O (green, yellow and red color respectively), e.g., green circles in (**A**) represent the percentage of cells that attained their first CP after growth in 0% D_2_O and division on agar plate with 0% D_2_O. The yellow circles in (**A**) represent the percentage of cells that attained their first CP after growth in 0% D_2_O and division on an agar plate with 70% D_2_O. The green circles in (**B**) represent the percentage of cells that attained their first CP after growth in 70% D_2_O and division on agar plate with 0% D_2_O. Yellow circles in (**B**) represent the percentage of cells that attained their first CP after growth in 70% D_2_O and division on agar plate with 70% D_2_O, etc. The percentage of cells that divided during experiments in their respective growth media is represented by a green dotted line for cultures grown in 0% D_2_O (**A**), a yellow dotted line for cultures grown in 70% D_2_O (**B**), and a red dotted line for cultures grown in 90% D_2_O (**C**). The horizontal dashed line represents 50% of cells in the culture and its intersection with individual CP lines gives midpoints of CP attainment.

To distinguish between the effects of D_2_O on growth and/or division, cultures of all experimental treatments were sampled throughout the cell cycle and small aliquots of all concentrations tested were transferred to agar plates and put into the dark until they divided. In this way, all growth processes were stopped, but the processes relating to progression of the cell cycle, DNA replication, nuclear and cell division were not affected. The proportion of cells that were able to divide in the dark, i.e., attain the CP is shown in Figure 5. To simplify the description of CP samples with regard to D_2_O concentrations in the growth medium and in the agar plates, their combinations (CP samples) will be designated according to Table 1. 

An increasing D_2_O concentration delayed CP attainment for both first and second CPs, ranging from 3.2 h and 8.4 h (first and second CP, respectively) in the 0–0 CP sample to 17.9 h and 25.4 h (first and second CP, respectively) in the 90–90 CP sample (compare Figure 5A,C). Moreover, when the cells were transferred to higher (or lower) concentrations of D_2_O upon darkening, the time required for CP attainment became longer (shorter) respectively, for all combinations (compare the positions of the green, yellow and red lines in Figure 5A–C). However, recovery upon transfer to a lower D_2_O concentration was limited in the 90–0 CP sample, as the percentage of cells passing both first and second CPs was significantly lower than in the 90–70 and 90–90 CP samples (Figure 5C). Interestingly, only about 40% of the cells were able to divide in both 70% (Figure 5B) and 90% D_2_O (Figure 5C), even though they seemed to be committed for division (Figure 5B,C). 

While the CP curves in Figure 5 give an overall summary of the D_2_O effect on CP attainment, quantification of such effects is summarized in Table 2. Section A (Table 2) quantifies the effect of D_2_O on cell cycle progression by comparing CP attainment in the reference 0–0 CP sample and the 70–70 and 90–90 CP samples. Increased D_2_O delayed attainment of both CPs in a dose dependent manner, the delay of the first and second CPs in 70% D_2_O represented 115% and 38% increases in time compared with the control, respectively, while in 90% D_2_O, the increase was 453% and 196% for first and second CPs, respectively. In Section B (Table 2), the conditions tested differed from their respective reference samples in the D_2_O content in the medium used during incubation in the dark, while the D_2_O content in each medium used for cultivation in the light was the same. Thus, the specific shift in attainment of the first and second CPs can be attributed mostly to the effect of D_2_O on division-related processes. For all combinations tested, the effect of transfer to medium with a different D_2_O content was dose dependent, i.e., with transfer to a higher D_2_O concentration, the time required to attain both CPs was longer and vice versa. Moreover, the effect of transfer on attainment of both CPs was not the same. In all cases, attainment of the first CP was shifted more (either prolonged or accelerated depending on the nature of the transfer) than the second CP. Interestingly, while the first CP in the 90–0 sample was accelerated by 89% in comparison with the 90–90 sample, the second CP was not reached in the culture (Figure 5C), and therefore the effect of transfer could not be assessed. 

### 3.5. Relative Timeline of Cell Cycle Events

**Figure 6:**
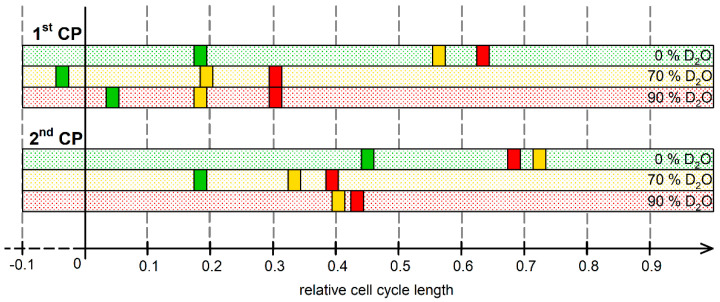
Midpoints of commitment point (CP) attainment in *C. reinhardtii* grown in D_2_O represented by relative cell cycle length. The three concentrations of D_2_O used for growth in the light are represented by three colored bars for the 1st CP and 2nd CP, respectively. Within each triplet, individual bars represent growth conditions as indicated on the right; green, yellow and red bars are assigned to growth in 0% D_2_O, 70% D_2_O and 90% D_2_O, respectively. Within each of the bars, midpoints of commitment point attainment after transfer to the dark to 0% D_2_O (green rectangles), 70% D_2_O (yellow rectangles) or 90% D_2_O (red rectangles) are displayed, indicating the relative incidence of the event within the cell cycle timeline. The midpoint of the second CP for the culture grown in 90% D_2_O and transferred to 0% D_2_O was not reached in the culture and therefore it is missing in the diagram (missing green rectangle in lowermost red bar).

So far, the progression of the cell cycle in deuterated and control cultures has been discussed in terms of individual CP attainments over time. This description is limited by one crucial fact—the cell cycles of the control and deuterated cultures varied significantly in their length. Therefore, information about the relative occurrence of individual cell cycle events within the cell cycle might not be obvious. To overcome this limitation, the timeline showing the occurrence of CP midpoints on the scale of relative cell cycle length was created (Figure 6). This representation allows us to compare the occurrence of such events between individual experimental treatments. For all cultures, both first and second CP attainments were affected by deuterium content during dark incubation in a dose-dependent manner (Figure 6; compare CP attainments within individual growth conditions). The shift in CP midpoint attainment caused by a change in deuterium content during dark incubation was less significant for the second CP than for the first. Furthermore, the timespan between second commitments of cells dividing in 70% D_2_O and in 90% D_2_O was significantly shorter than the timespan between first commitments of the same cultures.

## 4. Discussion

### 4.1. Growth and Accumulation of Energy-Storing Molecules in Media Containing D_2_O

Extensive replacement of protium by deuterium disrupts multiple physiological processes in the cell, including growth and division. The data presented show a decreased growth rate for cultures grown in 70% D_2_O or 90% D_2_O (Figure 2A–C). Similar findings were reported for *Chlorella* [16,35], *Scenedesmus* [36], *Dunaliella* [37], and *Spirulina* [38], as well as for *Chlamydomonas* itself [39]. Even though most reports show a concentration-dependent relationship between decreased growth rate of the culture and increased deuterium concentration, some studies report a stimulating effect of intermediate D_2_O concentrations (usually around 70% D_2_O) on observed growth rates [36]. Nevertheless, no such effect was observed in the data presented here. Division in deuterated cultures was accompanied by prolonged time periods (over 10 h) when daughter cells that arose from a single mother cell stayed together in a division cluster. Such structures, palmelloids, i.e., 4 to 16 cells surrounded by a cell wall, are known to originate from cell division without successive degradation of the mother cell wall. Formation of palmelloids is a stress-induced acclimation strategy in *Chlamydomonas* [40]. While growth rate of the cultures generally decreased with increasing D_2_O concentration, the opposite was true for cell size (Figure 2D). Enlargement of cells in medium rich in deuterium was previously reported in *Chlorella* [36,41] and *Scenedesmus* [42]. The increased cell size, together with lower cell counts (Figure 2C), suggests a decreased ability of *C. reinhardtii* to perform cell division in media containing 70% D_2_O or 90% D_2_O (Figure 4 and Figure 5). However, on the basis of these analyses, it is not possible to discriminate between the deuterium effect on cellular growth and cell division (see below). 

Apart from an increase in size, growth is characterized by the accumulation of energy-storing molecules. Although *C. reinhardtii* is generally considered a non-oleaginous alga because the carbon flux is primarily directed towards starch [43], lipids can accumulate under specific conditions, e.g., nitrogen or sulfur starvation [44], blockage in starch biosynthesis [45], salinity [46], heat [47] or high light [48] stress. Here, we report increased accumulation of both starch (Figure 3A) and neutral lipids (Figure 3B) in *C. reinhardtii* cultivated in growth medium enriched in deuterium. In a control culture without D_2_O, the starch content per cell reached a maximum at the twelfth hour of the cell cycle, representing 9.5 times the initial value, and then dropped gradually to nearly triple the initial value. The dynamics of cellular starch content reflects cell cycle progression in the control culture. The maximum value at the twelfth hour corresponds to the start of cell division in the culture (Figure 2C). With an increasing fraction of divided cells in the culture (Figure 5A, dotted line), starch was sequentially spent. Since cell division occurred in the light, the starch content did not drop to the initial value. No neutral lipid accumulation was detected in control cultures over the course of the cell cycle (Figure 3B). In both deuterated cultures, the pattern of starch and neutral lipid accumulation was different (Figure 3A,B), even though they arose from the same initial culture as controls and were grown at the same cell density. In 70% D_2_O, the cells accumulated about 60% more starch per cell than in the control culture, reaching the peak of starch content about four hours later than in the controls (Figure 3A). This corresponded to the start of cell division in the culture (Figure 5B, dotted line). Nevertheless, the decrease in starch content per cell caused by the proceeding cell division was not as significant as in the control culture, as it was maintained at nearly 60% of the maximal value until the end of the cell cycle (Figure 3A). Similar to the control, this can be partially explained by the fact that division was taking place in the light. Moreover, the proportion of dividing cells in 70% D_2_O was only 40% (Figure 5B, dotted line) suggesting that less starch was required for division. In this way, starch was (over)accumulated in such cultures. The neutral lipid content per cell increased, along with starch, in 70% D_2_O; however, no decrease that could be attributed to cell cycle progression was apparent (Figure 3B). In 90% D_2_O, the starch content per cell reached a plateau at around the 24^th^ hour of the cell cycle (i.e., 12 h later than the control culture) at a value comparable with the starch content in 70% D_2_O cultures (Figure 3A). Nevertheless, no decrease in net starch content per cell was observed in this treatment, as cell division in such cultures was spread over a long time, with an average of 40% of cells being able to complete their cell division (Figure 5C, dotted line). Thus, starch production within non-dividing (or already divided) cells could obscure starch consumption in the dividing cells. The (over)accumulation of starch may be a consequence of the greater effect of deuterium on cell division than on cellular growth (for more details see below). The neutral lipid content per cell in 90% D_2_O increased gradually to 8 times the initial value (Figure 3B). Notably, the onset of neutral lipid accumulation in both deuterated cultures was detected somewhat later than the onset of starch accumulation (Figure 3A,B). This might be due to the fact that oil synthesis in *Chlamydomonas* occurs only when a carbon supply exceeds the capacity for starch biosynthesis [43]. The same relationship between starch and lipid accumulation was shown for other green algae [49,50,51]. Deuterated organic compounds such as starch or lipids are highly valued in a wide range of applications [17,52]. They can be produced easily and economically by microalgae using the treatments described here. Understanding the mechanisms governing the (over)accumulation of deuterated starch and lipids can help in optimizing growth conditions for their biotechnological production. 

### 4.2. Cell Cycle Progression in Deuterated Cultures

Even though the effect of deuterium on various organisms has been studied since the 1930s [9,19,53], little is known about the extent to which deuterium affects cellular growth and division individually or in combination. Studies focused on microalgal growth in deuterium are often carried out using asynchronous cultures and growth of the culture is assessed by growth parameters such as dry weight, wet biomass, optical density, etc., which do not distinguish between growth in cell volume versus an increase in cell number [16,36]. Therefore, specific procedures that allow separation of the deuterium effect on cellular growth from its effect on cell division, such as CP assay in combination with transfers between D_2_O concentrations deployed in this study, were needed to address this question. The results of the CP assay revealed several consequences of deuteration on cell cycle progression of *Chlamyodomonas.* These ranged from straightforward and somewhat expected effects that could be directly observed on the cultures, to more complex effects that could only be discerned by a specific CP assay. The simple effects include prolongation of the cell cycle and effects on kinase activity of key cell cycle regulators, cyclin-dependent kinases (CDKs) (Figure 4). Prolongation of the cell cycle was evident from the course and timing of cell division in cultures with 0% D_2_O, 70% D_2_O and 90% D_2_O (Figure 5; compare the dotted lines). The length of cell cycle in 70% D_2_O was doubled, while in 90% D_2_O it was 3.3-fold longer than in the control culture. Prolongation of cell cycle is typical for suboptimal growth conditions, i.e., low light intensity [54] or suboptimal temperature [55], as well as application of antibiotics, such as chloramphenicol [56] or cycloheximide [57]. Moreover, the percentage of cells able to finish their cell division is considerably lower in deuterated cultures (Figure 5, dotted lines) and does not increase with prolonged cultivation (data not shown). The activity of CDKs, measured specifically as histone H1 kinase activity, in all experimental treatments, reached the main peak at the onset of cell division in the culture (Figure 4). The decreased kinase activity in deuterated cultures most probably reflects a lower proportion of cells undergoing cell division (Figure 4; compare the dotted lines in A, B, and C). In the control culture, there was an additional peak in histone H1 kinase activity around the fourth hour of the cell cycle (Figure 4A), i.e., at the midpoint of attaining the second commitment point (Figure 5A; green squares). Kinase activity corresponding to the first CP was not observed as the CP was attained very soon after the start of illumination and sampling intervals were too long to observe such a change (Figure 4A and Figure 5A, green circles). No additional peaks of histone H1 kinase activity were apparent in deuterated cultures, probably because of a combination of two factors: (1) the long time it took for the CP to be attained (Figure 5; compare the slope and total length of green commitment curves in A, yellow commitment curves in B and red commitment curves in C), and (2) the longer sampling intervals. Such results are in agreement with the role of cyclin-dependent kinases in the regulation of the cell cycle [27,58,59]. 

There were at least three phenomena that were observed specifically in the CP assay: (1) temporal shift of CP attainment, (2) difference in time-shifting of the first and second CP, and (3) the recovery from deuterium treatment. The temporal shift of CP attainment was observed in both deuterated cultures (Figure 5 and Figure 6) and comprised two distinct affects, with markedly different biological interpretations. The CPs in cultures grown in D_2_O without subsequent shift upon darkening (Figure 5B, yellow lines, and 5C, red lines) were clearly delayed in time compared to controls. However, when plotting midpoints of CP attainment on the timeline of relative cell cycle length (Figure 6), it became clear that the differences between the midpoints of CP attainments were rather minor (Figure 6, rectangles of the same color as the bar they are placed in). In 0%, 70% and 90% D_2_O, the first CP was attained at times corresponding to 18, 19 and 30% of the total cell cycle length, respectively, while the second CP was attained after times corresponding to 45, 33 and 43% of the total cell cycle length, respectively (Figure 6). This reflects the effect of D_2_O on both growth- and division-related processes, because both pre-commitment and post-commitment periods were prolonged in deuterated cultures. In the 90% D_2_O culture, the pre-commitment period was prolonged by up to 30% of the total cell cycle length (compared to 18% and 19% for the control and 70% D_2_O cultures, respectively). This suggests that growth-related processes characteristic for pre-commitment periods were more impaired, especially at the higher D_2_O concentration. From this perspective, the effect of deuterium resembled the effect of temperature on cell cycle progression, which also affects both pre- and post-commitment periods by similarly changing metabolic rates of both growth- and division-related processes [60]. Furthermore, this suggest that at 90% D_2_O, cellular growth is more affected than cell division, and it is thus more sensitive to the presence of deuterium. The second phenomenon observed in the CP assay was a different response of individual CPs to the treatment. Of the two CPs that were attained in the majority of conditions, the first CP was clearly more delayed than the second (Figure 5 and Figure 6). The midpoints of the first CP in 0, 70 and 90% D_2_O were attained after 3, 6.7 and 17.7 h, respectively. The midpoints of the second CP were attained after 8.3, 11.9 and 25.3 h, respectively. Thus, the prolongation of time required to attain the first CP in 70% and 90% D_2_O represented an approximately 2-fold and nearly 6-fold increase in comparison with the control, respectively. For the second CP, this increase represented an approximately 1.5-fold and 3-fold increase in comparison with the control culture, respectively. Such a difference in the effect of deuterium on individual commitments might suggest their distinctive role and importance in progression of the cell cycle of *Chlamydomonas*. This is in line with the concept of a single decisive CP, the first one, which changes the physiological status of the cell from one unable to divide in the dark to the one able to do so [23,61,62]. In this concept, the additional CPs are considered to be of lesser importance, as supported by the smaller effect of deuterium on the second CP. Apart from the different growth and cell cycle effects there was also an interesting phenomenon of recovery from deuterium treatment. The recovery potential seemed to decrease both with the deuterium concentration and the time spent in deuterium. The effect was particularly clearly seen on the decreased proportion of committed cells after transfer from 90% D_2_O to 0% D_2_O (Figure 5C, green lines). Even though in the early phases of the cell cycle, transfer to normal conditions seems to alleviate the stress caused by deuterium, prolonged cultivation in D_2_O followed by a shift to normal conditions resulted in a worse performance than in a culture deuterated for the whole cell cycle (Figure 5, compare the green and red lines). While a similar phenomenon of failed re-adaptation to normal conditions was also observed in *Chlorella*, it is probably not a general rule, as other microalgae, such as *Scenedesmus*, do not suffer any detrimental effects upon shift from D_2_O to H_2_O [53]. What needs to be further examined is whether the duration of exposure to D_2_O is the only critical factor determining the fate of cells upon transfer from deuterated to normal medium, as it was established that timing of the application matters significantly for other stress-inducing chemical agents [56,63].

### 4.3. Deuterium Affects Growth- and Division-Related Processes to Different Extents

The results of the modified CP assay, during which CP samples were transferred to different concentrations of D_2_O upon darkening, clearly showed that deuterium affects both growth- and division-related processes (Figure 5 and Figure 6, Table 2). The impairment of cellular growth is reflected by the relative prolongation of pre-commitment period in deuterated cultures (Figure 6). It is worth mentioning that while assessing that CP attainment is de facto dependent on cell division, it is unlikely that this would change the interpretation of the results for cellular growth impairment, as this is also supported by a delay in CDK activity in deuterated cultures (Figure 4). Division is affected by deuterium in a concentration-dependent manner (Figure 5 and Figure 6, Table 2, Section B). The effect of D_2_O being limited to cell division can be explained by “reinterpretation” of requirements for cell division after transfer, similarly to the temperature shift situation in *Desmodesmus quadricauda* [60], or by deterioration of processes linked specifically to nuclear or cellular division, e.g., dynamic stability of tubulin [64]. Furthermore, our results suggest that transfer of the cells to a lower D_2_O concentration upon darkening can accelerate the apparent attainment of CP and vice versa (Figure 5 and Figure 6). This is surprising, as it is widely accepted that the attainment of CP is set by growth via reaching the critical cell size. However, we see significant differences in both the timing and extent of CP attainment in cultures after transfers to lower deuterium concentrations. This contradicts the universal existence of a sizer that interrelates cell size with cell division, and it seems that cellular growth and the cell cycle simply correlate. Similar observations were made by manipulating the cultivation temperature of *Desmodesmus quadricauda*, where requirements for cell cycle progression were “reinterpreted” after a shift to a different temperature, as manifested by changed kinase activity of CDKs [60]. Further studies are needed to prove if such changes in CDK activity occur after a shift between D_2_O concentrations. 

## 5. Conclusions

We analyzed the effect of different concentrations of deuterium on growth and cell division in synchronous cultures of *Chlamydomonas reinhardtii*. We observed a concentration-dependent decrease in growth rates of deuterated cultures, which was accompanied by an increase in the net content of energy storage compounds such as starch and neutral lipids. We separated the deuterium effects on cellular growth and cell division by putting the cultures into the dark at different time-points, followed by a CP assay. The CP assay was further modified so that the cultures were transferred to different deuterium concentrations. This novel methodology allowed us to individually assess the effect of deuterium on cell growth and division. Deuteration was found to prolong the duration of the cell cycle in *Chlamydomonas*, change the pattern of kinase activity prior to cell division, and delay CP attainment (especially attainment of the first CP). Both growth- and division-related processes were negatively affected. These findings contribute to our knowledge of cell cycle regulation and coordination of growth and division. Moreover, an understanding of algal growth in a highly deuterated environment, its limitations and patterns of accumulation of energy storage molecules, might be used commercially to cheaply produce highly deuterated organic compounds.

## Figures and Tables

**Table 1:** Commitment point (CP) samples as defined by the combination of growth media and media used for dark incubation.

Growth Medium (% D_2_O)	Medium (% D_2_O) for Dark Incubation	CP Sample Name
0	0	0–0
	70	0–70
	90	0–90
70	0	70–0
	70	70–70
	90	70–90
90	0	90–0
	70	90–70
	90	90–90

**Table 2:** The effect of D_2_O on growth and cell division (section A), or specifically on cell division (section B) in *C. reinhardtii*. The effects are expressed as the proportional (%) change in the time required to attain the first or second commitment points (CP) in comparison with respective reference conditions. Both reference and tested conditions are described as in Table 1, i.e., 0–70 represents sample grown in media with 0% D_2_O, which was transferred to the dark to 70% D_2_O, etc. NA = effect not analyzed, as the CP was not attained by more than 30% of the cells.

	Reference Conditions	Tested Conditions	Time of 1st CP Midpoint Attainment (% Change)	Time of 2nd CP Midpoint Attainment (% Change)
A	0–0	70–70	+115	+38
		90–90	+453	+196
B	0–0	0–70	+216	+53
		0–90	+259	+45
	70–70	70–0	−120	−41
		70–90	+54	+20
	90–90	90–0	−89	NA
		90–70	−38	−5

## Data Availability

All data presented in this study are available within this article. There are no special databases associated with this manuscript.
